# Gitools: Analysis and Visualisation of Genomic Data Using Interactive Heat-Maps

**DOI:** 10.1371/journal.pone.0019541

**Published:** 2011-05-13

**Authors:** Christian Perez-Llamas, Nuria Lopez-Bigas

**Affiliations:** Research Unit on Biomedical Informatics, Department of Experimental and Health Sciences, University Pompeu Fabra, Barcelona, Spain; University of Leuven, Belgium

## Abstract

Intuitive visualization of data and results is very important in genomics, especially when many conditions are to be analyzed and compared. Heat-maps have proven very useful for the representation of biological data. Here we present Gitools (http://www.gitools.org), an open-source tool to perform analyses and visualize data and results as interactive heat-maps. Gitools contains data import systems from several sources (i.e. IntOGen, Biomart, KEGG, Gene Ontology), which facilitate the integration of novel data with previous knowledge.

## Introduction

High-throughput genomic experiments, which involve the usage of micro-arrays or next generation sequencing technologies, are used in many fields of molecular biology to discover genes implicated in particular processes. For better understanding of the results, global perspective analyses and intuitive visualization tools are needed in order to extract relevant information.

An important aspect in the interpretation of results from high-throughput genomic experiments is the integration of these data with previous biological knowledge. It is thus important to access large amount, yet structured biological knowledge, and to have tools that allow the integration and analysis of novel data in the context of this previous knowledge. Biomart [1] is a robust data integration system for large scale data querying used to provide easy access to a number of important biological databases such as Ensembl, ICGC data portal, ArrayExpress, COSMIC among many others (http://www.biomart.org). IntOGen [2] is a resource that integrates multidimensional oncogenomics data for the identification of genes and groups of genes (biological modules) involved in cancer development. KEGG [3] is an integrated database resource that provides biological information related to genes, pathways, diseases, compounds, etc.

Simple yet powerful approaches used in the analysis and integration of high-throughput biological data are; enrichment, clustering and correlation analyses among others. A number of tools are available that perform one or several of the above mentioned analyses; for example MeV [4], GenePatterns [5], ExpressionProfiler [6], geWorkbench [7], DAVID [8], Babelomics [9], GoMiner [10]–[11], ConceptGen [12], GSEA [13], EXPANDER [14] among many others.

In addition to the types of analysis to be performed over the data and the biological knowledge used to contextualize it, the visualization of data and results is very important, especially when many conditions are to be analyzed and compared. Heat-maps are graphical representations of data where values in a matrix are represented as colours. This has proven to be a very intuitive and useful way of visualizing biological data. Here we propose the use of interactive heat-maps to explore data and results. In an interactive heat-map every cell is associated to a set of values and annotations than can be visualized in colours and navigated interactively. A number of actions can be performed over the heat-map, which help to explore and interpret the results effectively, such as search, filter by value or label, cluster the heat-map, sort rows and columns by different criteria, move them freely, navigate from results heat-map to original data heat-map, etc.

We have developed Gitools, a desktop application for researchers with no necessary knowledge in bioinformatics that allows accessing many available biological databases, performing analyses following simple steps and visualising data using interactive heat-maps. Gitools is especially useful for cancer genomic analysis as it includes all the methods implemented for the Integrative OncoGenomics resource, IntOGen [2], and can import data directly from the IntOGen collation of experiments. However Gitools is generic enough and can be used for genomic analysis of any type and organism.

## Results and Discussion

**Figure 1:**
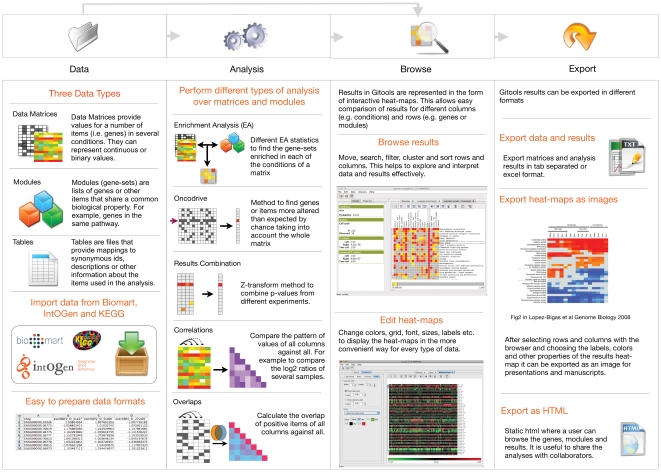
Main steps and features of Gitools. In the first step the data to be analyzed or visualized is prepared. This can be done by opening files that have been prepared externally or by importing the data from IntOGen, Biomart, Gene Ontology or KEGG databases. In the second step the user chooses which analysis to perform: enrichment, oncodrive, combination of p-values, correlations and overlaps. The parameters for the analysis are prompted using graphical wizards. In the third step the results are browsed and arranged in order to retrieve relevant information. In the fourth step the results are exported to tables, images and html.

The most common workflow in Gitools consists on four simple steps that can be followed in different ways (see Figure 1): (i) prepare data, (ii) perform analysis, (iii) browse data and results, and (iv) export tables, figures and reports.

### Prepare data

There are three data types that Gitools understands: matrices, modules and tables (see Figure 1). A matrix is a bi-dimensional structure in which for each dimension (row and column) there is a value. Modules (also known as gene-sets or concepts) are lists of genes or other biological elements sharing a common biological property. For example, all the genes involved in the cell cycle could form the module “cell cycle”. Gene Ontology terms and pathways are commonly used as modules. A table is a list of attributes, where each row is an element of the list and each column an attribute. Gitools supports many different file formats that represent these data types, which are easy to generate using any spreadsheet application like Excel or any text editor. Some of the supported file formats (i.e. GMX and GMT) are used by existing data repositories such as Molecular Signatures Database (MSigDB) [13], facilitating the used of gene-sets from this resource in Gitools.

Gitools allows retrieving data from several external data sources in the form of Gitools data types (matrices, modules and tables). The current version implements importers for Biomart [1], IntOGen [2], KEGG [3] and Gene Ontology [15]. The user can import matrices, modules and tables from those sources, and save them for posterior analysis and/or visualisation. One important advantage of Gitools data importers is that it allows the user to choose among many different types of gene identifiers for many different organisms. This makes Gitools a powerful application to work with genomic data independently of the organism or the type of gene identifiers.

IntOGen contains information of genes altered in hundreds of oncogenomics experiments. The alterations can be up-regulation and down-regulation for transcriptomic data, and gain and loss for copy number genomic data. Gitools can retrieve this information in the form of matrices and modules. Each of the available data types can be accessed at the level of individual experiments comparing tumour versus normal tissues among several samples, or at the level of combinations of experiments by tumour type. The ability to import data from IntOGen makes Gitools a valuable tool for oncogenomic data mining, and allows the comparison of user experimental data with hundred of already analysed oncogenomic experiments and combinations of experiments.

Gitools also allows retrieving data from Biomart systems. Biomart comprises many public databases as well as local installations that can be accessed from Gitools. One important database that can be accessed is Ensembl [16], it provides annotations for genes for many different organisms with cross-references for many types of gene identifiers. Further, the user can retrieve modules and annotations from different Ensembl releases. The generic importer from Biomart is very powerful as it allows users to obtain any type of data present in Biomart databases in the form of Gitools files. However it is also complex to use if one is not familiar with the data available in Biomart and its functioning. For that reason we have created dedicated importers to facilitate the import of the most commonly used modules and gene-sets such as Gene Ontology terms.

In addition we have created a dedicated importer for KEGG database, which includes the advantage of obtaining KEGG pathways for many organisms with many different types of gene identifiers.

### Perform analyses

The implemented methods in Gitools are enrichment, oncodrive, correlations, overlaps and combination of p-values (Figure 1).

The enrichment analysis is useful when the analysis at the level of genes is not enough to capture the full complexity of biological systems and a higher level view is required, for example at the level of pathways or biological processes. We have implemented different methods of hypothesis testing that can be used depending on the nature of the data. For matrices with real data (e.g. expression log2 ratios) we implement a z-score test using bootstrapping for mean or median estimation. For data measuring events (e.g. whether a gene has been found to be differentially expressed or not) we implement binomial test and Fisher's exact test. As many tests are performed at the same time, multiple test correction for the p-values can be applied.

The oncodrive analysis is a method to find genes or items more altered than expected by chance taking into account the whole matrix. It has been designed to identify genes that are significantly altered in sets of tumours (see IntOGen article [2] for more details). The combination of p-values is used to produce a combined test of significance across a set of experiments. When several experiments testing the same hypothesis are analysed, a natural question that arises is whether the combined evidence among them supports the hypothesis. However the individual experiments are often not directly compatible to produce a single large combined data set to be analysed together. Though, one can still produce a combined test of significance across a set of experiments. After computing p-values for each experiment independently we can integrate those results using the weighted Z-method [17]. This method is very convenient for integrating results obtained with other analyses like oncodrive or enrichment.

Other commonly used methods that we have implemented are correlation and overlap analysis. It is possible to perform correlations to compare patterns among matrix columns and rows, for example to compare patterns of expression among genes for different samples. It is also possible to analyse the overlap of positive elements between columns and rows in a binary matrix.

### Browse data and results

In Gitools results and matrices are represented as interactive heat-maps. This type of visualisation is very convenient because it allows having a wide view of the data as well as quick comparison of columns and rows. Several actions are available to perform over the heat-maps: sort by different criteria, display several annotations of rows and columns, search, filter the matrix by value or label, move rows and columns freely. Additionally one can perform clustering and calculate correlations and overlaps by columns or by rows. Every cell in the heat-map is associated with one or several values, for example in a heat-map resulting from an enrichment analysis every cell contains the statistical values derived from the analysis, such as the right, left and two-sided p-values, corrected p-values, observed and expected numbers, etc. By default a relevant value (e.g. right p-value for an enrichment analysis) is shown in colours in the heat-map, however the user can choose to show any associated value in the colour scale mode in each case. By clicking to each cell the user can see all the associated values in that cell (i.e. the details of the enrichment statistics for example). All these actions allow the user to interactively explore the data. Any matrix file can be opened as a heat-map without having to perform any analysis before, which allows the use of Gitools as a generic heat-map browser and visualizer.

To represent values of matrices and results we have implemented several colour scales, which are fully configurable for colours and significance level. In addition to changing the colour scale used for heat-maps there are other editable features that help to customize the heat-maps, such as the size of the cells, the grid lines, the font and the size for labels, etc. (see Figure 1).

### Export tables, figures and reports

The last step is to generate tables and figures that can be used in presentations or manuscripts as well as reports that can be shared with collaborators or published in Internet. Tables are exported as tabulated text format that can be easily opened with any spreadsheet editor like Excel, heat-maps and scales can be exported as images, and reports in HTML.

### Command line tools

Gitools is focused on being user friendly but also powerful enough to be used by advanced users in complex pipelines. This is why we have implemented some of the features available through the graphical interface using command line tools. Currently the following functions are implemented: gitools-convert to convert between file formats, gitools-enrichment to perform enrichment analysis, gitools-oncodrive to perform oncodrive analysis, gitools-correlation to perform correlations and gitools-overlaps to perform overlap analysis. Upgrades are scheduled for the near future.

### Comparing Gitools with other programs

Gitools has important unique features compared to other existing tools for genomic data analysis which make it a valuable addition and complement to many other software tools that permit the analysis and integration of novel data with previous knowledge, such as for example: MeV [4], GenePatterns [5], geWorkbench [7], DAVID [8], Babelomics [9], GoMiner [11] [10], ConceptGen [12], GSEA [13].

The most important distinctive feature of Gitools is the capacity of navigating data and results in the form of interactive heat-maps. This capacity makes Gitools useful for representing any matrix in the form of heat-map and navigate effectively the data on it even without doing any analysis. In addition, after any analysis performed in Gitools the results are shown as heat-maps in which each cell contain detailed information of the analysis results and it is possible to navigate between the results heat-map to the original data heat-map. Table S1 describes Gitools features in depicting and navigating heat-maps compared to other commonly used tools to depict heat-maps, namely MeV [4], GenePattern [5], Genesis [18], PageMan [19], CIMminer [20] and matrix2png [21].

**Figure 2:**
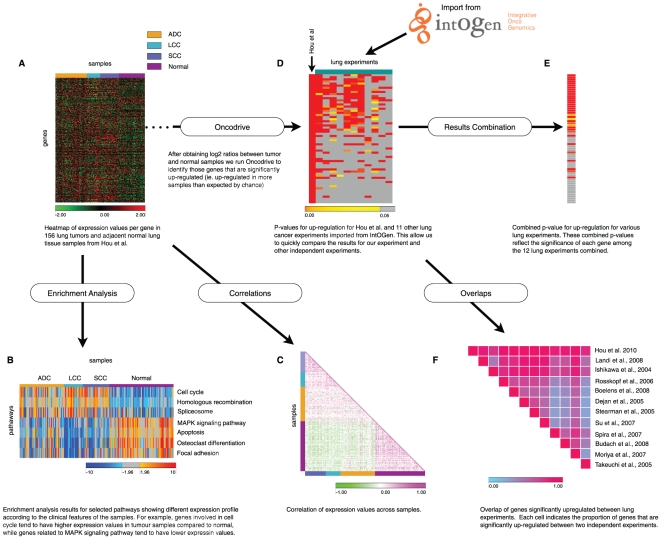
Case study. **A**. Expression matrix of a subset of probes for 156 samples classified as Squamous cell carcinoma (SCC), adenocarcinoma (ADC), large cell carcinoma (LCC) and adjacent normal lung tissue samples (normal) from Hou et al [25]. **B**. Heat-map of z-scores per pathway and sample resulting from an enrichment analysis using KEGG pathways as modules (only a selected subset of KEGG pathways are shown). **C**. Correlation of expression values per sample. **D**. Heat-map of p-values per gene indicating significant up-regulation for Hou et al experiment and 11 other lung tumor experiments imported from IntOGen. The heat-map shows the top significant genes in Hou et al experiment. **E**. One column heat-map depicting the combined p-value for up-regulation considering the 12 lung cancer experiments for the top significant genes in Hou et al. **F**. Result of the overlap analysis of genes significantly up-regulated in Hou et al and the other 11 lung cancer experiments from IntOGen.

Gitools currently performs 5 different types of analysis. Two of these analyses are unique to Gitools and not present in any other genomic software to our knowledge, Oncodrive and combination of p-values. On the other side there are many tools that perform enrichment analysis over a variety of gene sets and modules (see [22] for a review). The most important advantage of enrichment analysis in Gitools is that many conditions can be analysed at the same time and the results compared between them intuitively using interactive heat-maps. For example, in the case that many cancer transcriptomes (or various experimental conditions) are analyzed and we want to perform an enrichment analysis for modules (e.g. pathways) in each of them and compare the results for the different tumours, Gitools provides important advantages (see next section and Figure 2 for a real case study). First, to analyse all the samples (or conditions) a single run of the analysis is performed instead of one analysis run per sample as in most of the EA tools. Second, the results are shown in a heat-map, i.e. one column per sample and one row per module, which facilitate the comparison between samples and modules. Third, different actions, such as sorting, filtering, clustering, correlations and overlaps, can be performed over the results heat-map, which facilitate the exploration and interpretation of the results. Fourth, navigation from results heat-map to original data heat-map is possible, in the example proposed, this would allow to navigate from the module heat-map to a heat-map containing the genes in a particular module. Another advantage of enrichment analysis in Gitools includes the availability of many gene sets from generic and dedicated importers (i.e. Biomart, Gene Ontology, KEGG, IntOGen), which are available for many organism and many different types of gene identifiers. It is also important to note that Gitools understand various file formats for data matrices and gene sets. For example gmt and gmx file formats, which allows the use of the extensive collection of gene sets in MSigDB [13] in Gitools. Also tcm format (two column mapping), which is a common format used for gene sets, for example by DAVID knowledge base [8]. Table S2 describes similarities and differences of Gitools to other commonly used software to perform enrichment analysis (GSEA [13], DAVID [8], ConceptGene [23], ToppGene [24], Babelomics [9], Gominer [11] [10]).

### Case study

To illustrate the use of Gitools we have prepared one case study in which all 5 analysis methods currently available in Gitools are used (Figure 2). We used a data set containing 156 non-small cell lung carcinomas and adjacent normal lung tissue samples by Hou et al. [25].

We started with a matrix of expression values (median-centered log-intensity values divided by standard deviation) for the 156 samples (Figure 2A). We performed Zscore enrichment analysis for KEGG pathways for each sample of the dataset to identify pathways in which genes tend to have significantly higher or lower expression values (Figure 2B). For example, we found that genes involved in cell cycle, homologous recombination and genes that encode proteins of the spliceosome have higher expression values in the tumour samples compared to normal samples, however genes involved in MAPK signalling pathway, apoptosis, osteoclast differentiation and focal adhesion have significant lower expression values in tumour samples, specially in large cell carcinoma (LCC) subtype. We performed correlation analysis between expression values of different samples, finding higher similarities among samples with the same clinical classification (Figure 2C). This recapitulates the result obtained in the original manuscript describing this dataset [25]. We next obtained log2ratios between tumour and normal samples and used Oncodrive to identify genes that are significantly upregulated in this set of tumours (Figure 2D). Next we imported from IntOGen p-values for upregulation for other experiments analyzing lung tumours. We combined the p-values of the imported experiments and Hou et al experiment to identify genes that are significantly upregulated in lung cancer taking into account several lung cancer experiments (Figure 2E). We also compared the overlap of genes up-regulated in Hou et al experiment and the other lung cancer experiments imported from IntOGen (Figure 2F).

### Documentation and tutorials

The documentation on Gitools includes a user's guide, practical tutorials (including a tutorial to perform all the analysis presented in the Case Study section and shown in Figure 2), data and results examples and links to courses and presentations. It can be accessed either from the main web of Gitools at http://www.gitools.org or directly from http://help.gitools.org. All analysis wizards include a real biological example that allows the user to automatically fill the wizards with practical cases in biology to help exploring Gitools features step by step. Users are welcome to subscribe to the newsletter to stay updated about new releases or relevant events.

### Conclusions

The use of high-throughput techniques, such as micro-arrays and more recently sequencing techniques, is very common in genome research. The analysis of these data requires dedicated tools. Gitools types of analysis and visualization has shown to be very useful in a number of different types of projects ranging from cancer genomics [2], plant genomics [26], molecular biology [27] [28] [29], and evolutionary genomics [30]. For instance Gitools has been used for the integration of cancer genomics data [2], the study of RBP2 role in differentiation [27], a genomic analysis in melon [26] and the study of functional divergence in the evolution of *Homo sapiens* [30].

In summary, we have presented Gitools, a desktop application for genomics data analysis, which main features are the use of interactive heat-maps to navigate the data and results and the ready data import systems from several sources (i.e. Biomart, KEGG, IntOGen and Gene Ontology). These features are available to researchers without advanced knowledge on bioinformatics as well as to more experimented users that need to perform many of the operations available using the command line.

## Methods

### Access to external data sources

We have implemented a software module to access Biomart using RESTful web services independently of the location where Biomart is installed. This allows us to access not only to the Biomart Central Portal but also to any other Biomart portal whether it is a public database like Ensembl or a local installation. As a consequence we are able to access to different Ensembl releases as they are organised as different URL locations in the Ensembl server. There is an XML configuration file where more Biomart portals can be added. Based on this module we have implemented dedicated wizards to access this data for different common use cases, for example one to retrieve annotation tables and others to retrieve Gene Ontology and KEGG pathways gene sets. On the other hand IntOGen is accessed through customized web interfaces.

### Analysis methods

Gitools includes several analysis methods. For enrichment analysis, Z-score with bootstrapping, binomial test and Fisher's exact test are implemented. We implemented oncodrive to identify genes significantly altered in an experiment with a set of samples, see supplementary methods in [2]. For results combinations, we use the weighted Z-method [17]. For multiple test correction we implemented two methods, Bonferroni-Holm [31] and Benjamini-Hochberg FDR [32]. For clustering, we use the algorithms for hierarchical, K-means and Cobweb clustering implemented in Weka package [33]. For correlation analysis the method implemented is Pearson's correlation.

### Software organisation

Gitools is a portable desktop application programmed with Java, it requires Java 1.6 or above and it has been tested to work with Linux, Mac OS X and Windows systems. It has been developed following the best practises of software engineering. We apply well-known design patterns of software. It is highly modularised, separating core processes and user interface in different modules, allowing for better maintainability. The processes of building and testing are completely automatized using Maven [34]. We have made Gitools source code available through the subversion repository and we allow users to report bugs and suggestions using the Redmine project management web application [35] at https://bg.upf.edu/forge/projects/gitools. The documentation is maintained collaboratively by the group members using a wiki.

### Case study data pre-processing

We collected gene expression profiling data for tumors for Hou et al dataset [25] from Gene Expression Omnibus. CEL files were processed and normalized using the rma function in the “affy” package of R Bioconductor. The result of normalization is log2-transformed absolute reading. Each gene in the normalized expression matrix was median-centered across all the samples in the matrix and divided by the standard deviation. Log2 ratios between tumor and normal samples were calculated by subtracting the expression values (log2-transformed absolute reading) of each tumor sample by the mean expression value of all normal samples included in the dataset.

## Supporting Information

Table S1 Gitools features in depicting and navigating heat-maps compared to other commonly used tools to depict heat-maps.
(DOC)

Table S2 Similarities and differences of Gitools to other commonly used software to perform enrichment analysis.
(DOC)
