# Supplementary material for: Gitools: Analysis and Visualisation of Genomic Data Using Interactive Heat-Maps
Source: PLoS One. 2011 May 13;6(5):e19541. doi: 10.1371/journal.pone.0019541 (PMC3094337; doi:10.1371/journal.pone.0019541)
Supplement: Table S1 — Gitools features in depicting and navigating heat-maps compared to other commonly used tools to depict heat-maps. (DOC) [file pone.0019541.s001.doc]

**Table S1. Gitools features in depicting and navigating heat-maps compared to other commonly used tools to depict heat-maps.**

|  | **Gitools** | **MeV[4]** | **GenePattern heat-map visualizer module[5]** | **Genesis[18]** | **PageMan**  **[19]** | **CIMminer[20]** | **matrix2png**  **[21]** |
| --- | --- | --- | --- | --- | --- | --- | --- |
| **Interactivity** | | | | | | | |
| **Annotations for rows and columns** | Yes, rows and columns can have several descriptive attributes that can be used for visualization and operations | Yes, similar as in Gitools | Yes, similar as in Gitools | Only rows can have annotations and these have to be included in the original data file | No | No | No |
| **Actions over the heat-map** | - Move rows and columns freely  - Change visibility of rows and columns (shown or hidden)  - Filter by value or annotation  - Sort by value or annotation  - Simple clustering | - Move columns one by one  - Filter by values  - Sort by value or annotation  - Multiple clustering methods | - Sort by label | - Filter by values  - Sort by values and annotations  - Multiple clustering methods | - Highlight selected rows | - Clustering | None |
| **Navigate from results to data heat-maps** | Yes, the source data related to the elements selected in the results can be visualized in tables or new heat-maps. | No | No | No | No | No | No |
| **Multiple values per cell** | Yes, each cell can contain multiple values (e.g. all parameters and results of one analysis).  They can be visualized in an organized manner in the details panel when selecting a cell.  Any of those values can be used for colour representation in the heat-map. | Only one value per cell | Only one value per cell | Only one value per cell | Only one value per cell | Only one value per cell | Only one value per cell |
| **Edit capabilities** | | | | | | | |
| **Interactive edition** | Yes, the heat-map is an editable document, not a static image. Each edition change over the heat-map is immediately reflected. When the user is satisfied with the appearance of the heat-map the final image can be exported. | Yes, similar to Gitools | Yes, similar to Gitools | Yes, similar to Gitools | Yes, similar to Gitools | No, the heat-map is a static image. To make edit changes the user has to create a new image with new parameters. | No, similar to CIMminer |
| **Types of colour scales** | Continuous single and double gradient, p-value, z-score and binary discretized | Continuous single and double gradient | Continuous and discretized double gradient | Continuous single, double or multi gradient | Continuous double gradient | Continuous four gradient | Continuous double gradient and discrete scales. |
| **Scales that discriminate between significant and non-significant values** | Yes, p-value and z-score scales | No | No | No | No | No | No |
| **Scale are configurable for colours and numeric ranges** | Yes | Yes | Yes | Yes | Only predefined colour schemes | Yes | Yes |
| **Appearance configuration** | - Grid (visibility, size and colour)  - Cell size  - Headers (size, font and colours) | - Grid (visibility)  - Cell size  - Headers (font) | - Grid (visibility)  - Cell size  - Headers (labels visibility) | - Grid (visibility)  - Cell size  - Headers (width, label visibility)  - Standard deviation and average bars  - Indicate absolute maximum | - Grid (size)  - Cell size  - Headers (font size)  - Border | - Headers (font size, labels and clustering tree location) | - Grid (visibility)  - Cell size and shape  - Headers (labels location) |
| **Export** | | | | | | | |
| **Export heat-map as image** | Yes | Yes | Yes | Yes | Yes | Yes | Yes |
| **Export reports** | Html tables for the source data  and results | No | No | No | No | No | No |
| **Export data** | Yes | Yes | Yes | Yes | No | No | No |
