# Supplementary material for: Gitools: Analysis and Visualisation of Genomic Data Using Interactive Heat-Maps
Source: PLoS One. 2011 May 13;6(5):e19541. doi: 10.1371/journal.pone.0019541 (PMC3094337; doi:10.1371/journal.pone.0019541)
Supplement: Table S2 — Similarities and differences of Gitools to other commonly used software to perform enrichment analysis. (DOC) [file pone.0019541.s002.doc]

**Table S2. Similarities and differences of Gitools to other commonly used software to perform enrichment analysis**

|  | **Gitools** | **GSEA[13]** | **DAVID[8]** | **ConceptGene[23]** | **ToppGene[24]** | **Babelomics[9]** | **Gominer[10][11]** |
| --- | --- | --- | --- | --- | --- | --- | --- |
| **Input Data** | | | | | | | |
| **Source of gene sets** | Direct import of gene sets using dedicated importers from Biomart, IntOGen, KEGG and GO databases. KEGG, GO and Biomart genesets are available for all organisms accessible in those databases. Many gene identifiers are supported, all in the cross references of Ensembl. | Prebuilt gene set collection (MSigDB) mostly from human and some for other organisms. Gene identifiers available are gene symbols and entrez gene ids. | Prebuilt gene sets collection from many organism  (DAVID knowledgebase). Gene identifiers and annotation contents are clustered and centralized by one single index, DAVID Gene Identifiers. This allows the use of many different gene identifiers | Prebuilt gene set collection for human, mouse and rat. Gene identifiers supported are gene symbols and entrez gene ids. | Prebuilt gene set collection for human. Gene symbol, Entrez ID, Ensembl ID, RefSeq, Uniprot gene ids are accepted. | Prebuilt gene set collection for 9 organisms. Internal Id converter to many different gene identifiers. | Gene Ontology terms for 12 organisms. Various gene identifiers supported. |
| **Possibility to analyse your own gene sets** | Yes. Gene sets are accepted in various easy to prepare formats. gmx and gmt formats are useful to analyze MSigDB gene sets and tcm format is useful to analyze gene sets from DAVID knowledgebase. | Yes. Gene sets are accepted in various easy to prepare formats (e.g. gmx and gmt formats) | No | Yes. One gene set at a time can be included by pasting a gene list in the web. | No | Yes | No |
| **Possibility to analyse many conditions with one run** | Yes | No | No | No | No | No | Yes |
| **Statistics** | | | | | | | |
| **Enrichment Analysis Statistics for continuous values** | z-test with permutations | Kolmogorov–Smirnov rank statistics | None | None | None | Fatiscan[8] | None |
| **Enrichment Analysis Statistics for lists of genes** | Binomial distribution and Fisher’s exact | None | Modified Fisher’s exact - EASE | Modified Fisher’s exact - EASE | Hypergeometric distribution | Fisher’s exact | Fisher’s exact |
| **Platform** | | | | | | | |
| **Platform** | Java | Java / R | Web | Web | Web | Web | Web / Java |
| **Command line options** | Yes | Yes | No | No | No | No | Yes |
| **Results exploration** | | | | | | | |
| **Comparison of EA results for various conditions using heat-maps** | Yes | No | No | No | No | No | Yes |
| **Perform actions over the heat-map, such as sorting, filtering, correlations, clustering, etc.** | Yes | No | No | No | No | No | No |
| **Navigate from results heat-map to data heat-map** | Yes | No | No | No | No | No | No |
